# Recovery at the Clubhouse: challenge, responsibility and growing into a role

**DOI:** 10.1080/17482631.2021.1938957

**Published:** 2021-06-15

**Authors:** Orsolya Reka Fekete, Eva Langeland, Torill M. B. Larsen, Larry Davidson, Liv Grethe Kinn

**Affiliations:** a Faculty of Health and Social Sciences, Western Norway University of Applied Sciences, Bergen, Norway; b Department of Health Promotion and Development, Faculty of Psychology, University of Bergen, Bergen, Norway; c Department of Health and Caring Sciences, Faculty of Health and Social Sciences, Western Norway University of Applied Sciences, Bergen, Norway; d Yale Program for Recovery and Community Health, Yale University, New Haven, CT, USA; e Department of Welfare and Participation, Faculty of Health and Social Sciences, Western Norway University of Applied Sciences, Bergen, Norway

**Keywords:** Clubhouse model, mental illness, psychosocial rehabilitation, recovery, salutogenesis

## Abstract

**Purpose:** To explore how people with mental illness experience recovery in the Clubhouse context, and which ingredients of the model they find active in promoting recovery.

**Methods:** Hermeneutic–phenomenological design. Individual, semi-structured interviews with 18 Norwegian Clubhouse members. Systematic text condensation was used in analysis.

**Results:** Three main themes emerged: “Balancing unlimited support with meeting challenges”, with two sub-themes: “Unlimited membership: space for self-agency or hindering development?” and “Becoming a Clubhouse member: concerns and positive experiences”. The second main theme was: “Learning how to build new skills and roles in the community”. The third main theme was: “Getting better through and for work”, with two sub-themes: “Work at the Clubhouse as a means to recovery” and “Preparing for a working life in society”. Overall, participants experienced improved mental and social wellbeing and work readiness.

**Conclusions:** Recovery in the Clubhouse context requires members’ personal initiative, thus people having poor mental health might struggle with utilizing the Clubhouse. However, participants reported that lack of challenges within the community thwarted their recovery. Based on Salutogenesis, conscious application of challenge in Clubhouse activities might enhance members’ recovery. Furthermore, participants’ all-round involvement in their recovery journeys suggests the importance of shared decision-making in recovery-oriented services.

## Introduction

Empirical evidence has been mounting for several decades that mental illness is not a condition that inevitably deteriorates and that it is possible to recover from it (Davidson, 2003; Langeland et al., 2007; Leamy et al., 2011). Consequently, the recovery paradigm has become the guiding principle in global mental health (Anthony & Mizock, 2014; Le Boutillier et al., 2011; Ministry of Labour & Ministry of Health and Care Services/Arbeidsdepartementet & Helse- og omsorgsdepartementet, 2013; Norwegian Directorate for Health and Social Affairs/Sosial- og helsedirektoratet, 2005; World Health Organization [WHO], 2013).

Recovery has been described as a deeply personal, unique and transformative process, during which the person in recovery redefines her- or himself and several aspects of their lives, with the hope of living a satisfying life despite struggling with a mental illness (Anthony, 1993; Deegan, 2002). This is compatible with the theory of salutogenesis and the field of psychosocial rehabilitation (PSR), both of which emphasize that every person, at all times, has a healthy aspect to build upon (Anthony & Liberman, 1986; Antonovsky, 1979, 1987b). Furthermore, the recovery and salutogenesis literature highlights the distinction between “recovery from” mental illness, a biomedical approach with a focus on symptom management and treatment, versus “being in recovery”, an approach in which recovery is described as a continuous active adaptation process in order to live a satisfying life in the face of a mental illness (Antonovsky, 1979, 1987b; Davidson, 2003; Davidson & Roe, 2007). According to the theory of salutogenesis, three personal attitudes might enhance the healing of a person in recovery (Langeland et al., 2007). First, it is necessary that the individual develop a more constructive identity other than that of a patient suffering from mental illness. Second, having confidence or being proactive in meeting one’s challenges can promote processes of recovery. Third, since health incorporates multiple aspects of wellbeing, the individual’s processes of being in recovery must take account of these, including physical, mental, social and spiritual dimensions (Langeland et al., 2007).

Noticeably, the recovery literature also underlines the social nature of recovery, pointing out that the social dimension is inherent in the recovery process (Mezzina et al., 2006; Topor et al., 2020). According to studies, the social surroundings might serve as a source of support and point of cultural reference for the individual, and an environment to exercise the self-agency one develops in their process of recovery (Mezzina et al., 2006; Topor et al., 2011, 2020). Therefore, high quality of social support in one’s social environment is crucial as a part of recovery process (Langeland et al., 2016; Topor et al., 2020), and being a member of a Clubhouse might represent such a social environment.

As each person has a unique recovery process, the patient/consumer’s involvement in and active adaptation of all aspects of service delivery/treatment are crucial (Antonovsky, 1979, 1987b; Le Boutillier et al., 2011; Davidson et al., 2017). Furthermore, because recovery is a non-linear process, recovery-oriented services should ideally have a flexible and individualized approach (Le Boutillier et al., 2011). Accordingly, recovery-oriented services are intended to provide support for the individual in achieving their personal goals (Le Boutillier et al., 2011; Davidson et al., 2007). Research suggests (Le Boutillier et al., 2011; Davidson, 2016; Davidson et al., 2017; Dixon et al., 2016; Fekete, Langeland et al., 2020; Langeland et al., 2007) that the engagement of the person in recovery in all aspects of care, or in shared decision-making (SDM), has a positive impact on outcomes and increases wellbeing. However, studies indicate that personalized care and SDM are not evident in practice (Davidson, 2016; Dixon et al., 2016; Oute et al., 2018). A possible reason for this is that service providers have difficulty in ascertaining consumer/patient preferences (Woltmann & Whitley, 2010), a factor that has been associated with a higher level of involvement of the consumer in SDM (Fukui et al., 2013).

We suggest that gaining an understanding of how people with mental illness experience their recovery process in the context of an evidence-based and recovery-oriented programme, such as the Clubhouse model (McKay et al., 2016; Raeburn et al., 2013), might help to develop a better insight into consumer/patient preferences. Apparently, there is a wide range of research related to the Clubhouse model, still, we could not identify research exploring members’ experiences of vocational and social recovery processes in the Clubhouse community, which is the aim of the present study.

### The context: the Clubhouse model

The Clubhouse model is a pioneer among PSR programmes (McKay et al., 2016; Raeburn et al., 2013), with the first Clubhouse, Fountain House having been established in 1948 in New York. Today, 300 Clubhouses operate in 38 countries (Clubhouse International, 2020). The model is regulated by the International Standards for Clubhouse Programmes, which also “serve as a ‘bill of rights’ for members and a code of ethics for staff, board and administrators” (Clubhouse International, 2018, Preamble) and provide a basis for quality assessment.

The programme offers community experience and useful activity for people with a history of mental illness (Norman, 2006; Raeburn et al., 2013). The principles and terminology of the model reflect its non-clinical nature. Thus, participants in the programme are referred to as member instead of user or patient, and participation is strictly voluntary and free for life (Battin et al., 2016; McKay et al., 2016; Propst, 1997; Raeburn et al., 2013). The programme offers their members community experience and possibilities of participation in useful activity (Norman, 2006; Raeburn et al., 2013). The basic intervention of the model is the work-ordered day (WOD), a workday following customary working hours in society. According to the Clubhouse model, staff and members are intended to collaborate side by side as equals on doing tasks related to operating the Clubhouse, from cleaning bathrooms through to making lunch, writing grants and planning programmes (Battin et al., 2016; McKay et al., 2016; Raeburn et al., 2013). Notably, the staff are not expected to act as service providers or care workers; their main task is to engage members in activities and assist in their inclusion in the Clubhouse community (Clubhouse International, 2018). In addition, Clubhouses offer support services for their members such as employment support, education support and access to housing and entitlements. It is not necessarily the staff who provide these services, but possibly a fellow member or a task group within the Clubhouse community (Biegel et al., 2013; Coniglio et al., 2012).

### Previous Clubhouse research

Previous studies on the Clubhouse model have revealed outcomes that are arguably a part of the members’ recovery journeys. For example, results from several quantitative studies indicate that participation in the WOD reduced relapse (Beard et al., 1978, 1963; Delaney, 1998), improved psychopathology (Tsang et al., 2010), quality of life (Accordino & Herbert, 2000; Tsang et al., 2010; Unger et al., 2002) and work readiness, and increased employment duration (Bonsaksen et al., 2016; Schonebaum & Boyd, 2012). In addition, a meta-ethnography of 16 qualitative studies of staff’s, members’ and families’ experiences of the Clubhouse model (Kinn, Tanaka et al., 2018) revealed that individuals’ recovery journey within the community can be described according to the themes “stepping out of limiting realities”, “anchoring” and “flourishing”. Other qualitative studies, such as that of Tanaka and Davidson (2015a), revealed that the WOD improved members’ autonomy, a common recovery goal, through developing work skills and providing respite and a sense of accomplishment. Moreover, Mutschler et al. (2018) findings indicate that participating at the Clubhouse helped members to feel better and at peace, to develop a sense of personhood beyond their identity as a patient and to acquire social, work related, and daily life skills.

Other qualitative studies relating to the Clubhouse model concerns the relationships and social networks of Clubhouse members, as these factors are strongly associated with recovery. Indeed, studies (Pernice-Duca & Onaga, 2009; Tanaka & Davidson, 2015b) have found that having reciprocal relationships at the Clubhouse indicates progress in recovery. Further research has claimed that members consider the Clubhouse community as an opportunity to (re)build their social network (Carolan et al., 2011), and have a strong emotional connection with it, considering their Clubhouse community as a “substitute family” (Fekete, Langeland et al., 2020; Pernice-Duca, 2008).

## Methods

### Study design

A qualitative, hermeneutic-phenomenological design was chosen to help to understand and account for people’s experiences on the subject matter and how they construct meaning in their ordinary life world (Dowling, 2007; Gadamer, 2013; Laverty, 2003). Based on this approach, we sought a description of how participants constructed meaning of their experiences regarding the phenomenon of “recovery in the Clubhouse context” and interpret the findings in light of the research questions, by exploring the individual accounts in an inductive and iterative manner (Dowling, 2007; Gadamer, 2013; Kvale et al., 2015; Laverty, 2003). This study is part of the project “What is it like to be a Clubhouse member?”—Qualitative studies in a Norwegian context.

### Participants and sampling

The study applied purposeful sampling, where being an established Clubhouse member was the criterion for inclusion. Members from three accredited Clubhouses, two in central Norway and one on the west coast of Norway, agreed to participate. An invitation letter with information about the project was made available to the members. Participants were also informed of their rights, such as the right to anonymity and the right to withdrawal at any time without consequences.

Participants gave their informed consent in writing before the interviews. Altogether 18 Clubhouse members, consisting of five women and thirteen men between the ages of 27 and 75, contributed with interviews, which had an average length of 50 minutes.

### Data collection

For the convenience of the participants, the researcher visited their Clubhouses and conducted the interviews locally, in a separate office at each participant’s Clubhouse with only the researcher and participant present to ensure privacy and anonymity.

Individual, semi-structured interviews were conducted during the data collection. An interview guide was used to ensure the consistency of the interviews, including questions such as: “Can you describe what it was like to come here in the beginning?”, “What do you experience to be helpful to your recovery at the Clubhouse?”, “Which activities at the Clubhouse do you find useful for entering the labour market?”, “What are your recovery goals and how does the Clubhouse help you in achieving these?” and “How has your life changed since you joined the Clubhouse?” All interviews were audio recorded, and the researcher took notes to assist in the subsequent analysis.

### Data analysis

Audio records were transcribed verbatim, partly by the first author and partly by a contractor. The method of analysis was systematic text condensation (Malterud, 2012), a four-step method. Inspired by Giorgi’s phenomenological method, STC is a descriptive and explorative method (Malterud, 2012), following a four-step procedure for analysis, which lent itself for the project’s hermeneutic-phenomenological design. The first step, which was conducted by all authors, was to identify preliminary themes that emerged spontaneously from the material. Taking these preliminary themes as a starting point in step 2, meaning units were identified in the original text, decontextualized from their original context, sorted by codes and classified, which resulted in the final themes. Subsequently, in step 3, the extracted meaning units were rewritten as a continuous text in the first person for each theme (condensates). Finally, in step 4, the condensates were re-narrated in third-person format and recontextualised in order to “elucidate the research question” (Malterud, 2012, p. 800). As a result, an analytic text was prepared presenting the major ideas within the material concerning the phenomenon in question and illustrated by excerpts from the original interviews to represent the voices of participants (Malterud, 2012). During the analytical process, the results from steps 2 to 4 were continuously reconsidered in order to ensure the credibility of the data (Cope, 2014). The final findings were validated against the original transcripts and were reviewed and accepted by all of the authors (Malterud, 2012).

### Ethics

The researcher had no personal affiliation with any of the participating Clubhouses. Procedures of cooperation were established in order to ensure the quality of the study process. These included regular meetings of the research group and the provision of continuous feedback to the first author, with emphasis on reflexivity (Probst, 2015).

## Results

Three main themes emerged from the interviews regarding the participants’ experiences of change and the factors promoting it in the Clubhouse environment, and sub-themes were developed under two of the main themes in order to provide clarity of the major topics building these main themes up. The first main theme was “Balancing unlimited support with meeting challenges”, with the two sub-themes “Unlimited membership: space for self-agency or hindering development?” and “Becoming a Clubhouse member: concerns and positive experiences”. The second main theme was “Learning how to build new skills and roles in the community”. Finally, the third main theme was “Getting better through and for work”. This main theme was also built up by two sub-themes: “Work at the Clubhouse as a means to recovery” and “Preparing for a working life in society”. Table I shows an overview of the main- and subthemes we have developed.

**Table I.** Main themes and subthemes

Main themes	Sub-themes
Balancing unlimited support with meeting challenges	Unlimited membership: space for self-agency or hindering development?
	Becoming a Clubhouse member: concerns and positive experiences
Learning how to build new skills and roles in the community	
Getting better through and for work	Work at the Clubhouse as a means to recovery
	Preparing for a working life in society

### Balancing unlimited support with meeting challenges

Participants’ experiences of recovery at the Clubhouse seemed to be a balancing act between security and challenge, self-agency and support, described by the sub-themes below.

#### Unlimited membership: space for self-agency or hindering development?

Notably, every participant spoke appreciatively about how the free, lifelong membership the Clubhouse offered gave them time to recover. They linked this to positive experiences, such as being offered unlimited time to get better without any pressure and being ensured a stable support system to fall back on. As Lucas said:

I don’t know what it will be like then (when I get a job), but, uhm, I think that in any case the Clubhouse will be here for support if I need (…) if it is not working out very well, so I could, instead of getting sick leave and sitting at home, so I can come here and do … use the Clubhouse. Or I could get help with making the job work if I needed it.

Despite the leeway provided by unlimited membership, most of the participants seemed to agree that one must make the most of this time. For example, some voiced concern about settling into the Clubhouse community so well that one would hesitate to pursue goals outside of it. Mathias was one of those, saying: “But this is … unlimited time, which makes you … makes you relaxed. Uhm, hope not too much, because one would want to go out to work … ” Still, most of the participants appreciated the opportunity of having the freedom to determine the pace of their own recovery, deciding their recovery goals and choosing the activities they wanted to partake in.

Commitment to sobriety seemed to be another important decision to make in order to participate at the Clubhouse. Some participants said that it created a more pleasant environment, while other participants said it motivated them to become drug-free. Notably, a participant credited this measure with enhancing the recovery focus in the Clubhouse community:

Mantra number one in the house is being drug-free when you are here, in any case, no matter what. And I feel that if we, like, let it slide that people can (come when they are under the influence) … it would kind of lower the bar too much, because this is actually a place where people have taken the decision that they want to be healthier. (Thomas)

Many of the participants reported that taking their recovery into their own hands and asking for help seemed to be the most basic form of taking initiative. Participants mentioned a range of issues they might ask for help with, such as contacting official entities such as one’s GP or the Labour and Social Services Agency or writing CVs and official correspondence. At the same time, many of the participants noted that help was readily available at the Clubhouse:

Very easy (to get help). You do go to for example … like the way I did, you go to a staff member and say … now I would like to do this, and I need a little help with it … and so you get that help. If that person there cannot help you, so you maybe try to find someone who can help … (Emma)

However, some of the participants described issues connected with receiving help from staff, suggesting that receiving help might also be dependent on one’s readiness to take control. For example, according Theodor “those who are more uninhibited get help, and those who are in a way a bit slow, they fall into oblivion.”

Still, participants seemed to turn to staff more than their peers to provide them with support. However, some participants emphasized the risk of staff solving members’ problems rather than helping them to meet challenges themselves. However, others, such as Oscar, described being able to grow from meeting challenges posed by staff:

So, after half a year, and then became … Then I thought, then I was still, in a way … Had extremely low self-esteem, so, no, I did not want to do the whiteboard presentation (task allocation at the unit meeting), as I said to a staff member. But then she went: “Well, you can learn. You do this there.” “No, no, no.” “But yes!” And then she persuaded me to do it then. And then things went pretty well … (Even) if that’s not the case (i.e. success), right? So, you have to be really … You should try, okay, cut out complaining and stuff, and focus on resources and stuff. And I really did.

#### Becoming a Clubhouse member: concerns and positive experiences

Owing to the fact that participants described making conscious choices and taking initiative as key elements in their recovery processes, it seemed to be the general experience that the Clubhouse would best suit people who are prepared enough to make these efforts. For example Anna, described herself as not being ready for the Clubhouse the first time she tried to join:

I was at the Clubhouse maybe a year ago. I was there three times with my therapist, who recommended it (the Clubhouse) to me. But then I only came here those three times, because I was … I think I didn’t know why … not totally why I should be here, so I wasn’t ready. Then a year passed, and I became super ill, and so … well, in the hospital they are really aware of the Clubhouse. They knew that this here is really good, so they tried to get me in here. So I came here, and so I met again one of the staff members who works here, I knew some of the people here a little, right, and so I felt that this is my only chance to … to get a … get an … everyday, which … well with routines.

Opposite of Anna’s experiences, other participants who had a positive community experience, mentioned becoming members at their first encounter with the Clubhouse, such as Emil, who said “ … it (the Clubhouse) was at once very inclusive. I felt myself … I was very welcome, but not just in the beginning. (…) I was shocked that everybody said hi.” Participants like Emil reported to find a suitable opportunity in the Clubhouse; both as place to socialize, and as an alternative to other mental health services, which they had not provided them with suitable results.

However, a few participants said that they had found the introduction phase confusing and insecure and, as a result, they considered quitting in the early stages of their membership. As Lucas said:

I was frustrated then, because I didn’t quite know what to do, and, and no one saw it either, so … I was about to stop coming here. (…) And it was a little complicated, it was … People thought I needed help all the time. That I didn’t understand what to do. But … Yes, I talked to a staff member and then, uhm, she thought I was a good fit for accounting. And that … So, after that, I got some training, uhm, in the routines there and … Yeah, ever since then I’ve been dealing with the accounting.

Others described taking a careful approach to settling into the Clubhouse because of anxiety arising from previous experiences of exclusion and stigmatization in other services.

In addition, some participants suggested a level of difficulties with including everyone equally into the Clubhouse community. These participants talked about feelings of discomfort facing peers whose behaviour was dominated by their mental illness. These members with apparent symptoms were perceived to not fit in the community because they were not ready to offer support for others, thus contribute to the community effort or were perceived as draining the energy of others. Axel described it as such “I miss a normal workplace sometimes. Where you might not have to deal with sick people (…) It can be tiring.”

### Learning how to build new skills and roles in the community

All of the participants said that one of the means of improving their everyday coping and wellbeing was participation and social interaction in the Clubhouse community. They reported that the Clubhouse environment was warm and welcoming, which provided them with a safe space for social interaction. However, settling into the community was described as a difficult process of learning the system of meetings, tasks and getting familiar with the status quo between members and staff.

Getting used to the Clubhouse seemed to bring with it the task of defining relationships. For example, some participants described developing relationships in which they could open up by discussing their challenges and asking for or listening to other members’ opinions about them. However, some of the participants also said they had to establish boundaries so as not to exhaust themselves by socializing with those whom they were not particularly drawn to. Still, many of the participants saw the interactions with their peers as an important part of their personal growth process. Several participants, such as Emma, described positive changes in their social capacities as a result of social interactions at the Clubhouse:

So I … I actually learnt quite quickly when I started collaborating with others that I could be quite harsh, but … and when I thought when I was home and was only for myself, sort of, but … then you got that feedback … not so straight maybe, but you realised that here I have to do something.

Another important topic in the community seemed to be discussing one’s mental health challenges with both fellow members and staff, which is demonstrative of a feeling of security. Notably, the shared experience of mental illness seemed to be a unifying issue for members, and it gave them an opportunity to provide a kind of nurturance to each other that the staff could not give. Maya described this as follows:

It is also cool to be able to mean something for someone in that situation. To be able to be with the tours and be able to support them and say, “You’ll get over this, this is just a bump” and like “I’ve been there myself”, and, well … And to only be able to say that sentence, that you yourself have been hospitalised, uhm, that, it established a kind of trust that, uhm, maybe those who are staff members cannot …

Many participants also credited their symptomatic improvement to their Clubhouse membership. In fact, some participants said that they purposefully used the Clubhouse as therapy, such a form of exposure therapy for social anxiety. Yet another participant, Thomas, chose the Clubhouse over another form of treatment:

I had been ill for a long time, eh, and I thought, “What will it take for me to recover even faster? What will it take for me to have a much faster recovery process?” (…) So my therapist said: “I cannot do anything more for you, either medically or conversationally, but get yourself into group therapy.” But much of what I was able to imagine or that was promised to happen in group therapy, has, has … is actually happening here at the Clubhouse. (…) Uhm, so I feel like this has worked as a treatment for me.

Every participant commented on different aspects of the positive social influence of belonging to a Clubhouse. As one of them put it, the Clubhouse was a place where one could “exercise their social muscle.” Indeed, several participants said that the Clubhouse was an arena for honing their social skills, especially after a long bout of isolation.

Uhm, there is a lot of opportunity to socialise here, anyway. To talk to people and make friendships and maintain them. Uhm, so I don’t get stage fright. If you have spent too much time alone then it is scary to go out. So, you break that (pattern) in a way. (Axel)

Moreover, many of the participants underscored the importance of having a community that was open and available to them no matter the circumstances. For instance, one participant noted that this was the first place in her life where she felt accepted, and several participants reported having built up a sizable part, if not all, of their social network through the Clubhouse.

### Getting better through and for work

Work was a central topic of the interviews, emerging in two subthemes: the meaningful work one did at the Clubhouse as a means to propel the recovery process, and obtaining and keeping work in the labour market as a recovery goal.

#### Work at the Clubhouse as a means to recovery

In terms of work as a means, several participants said that it was important for them to be able to do meaningful tasks for the Clubhouse community because it helped them not to ruminate over their problems and health issues. Accordingly, many participants reported feeling healthier by working at the Clubhouse, merely by shifting their focus from their illness to something positive. One of the participants, Maya, described it by saying “you must fill your day with something meaningful. (…) It doesn’t have to be much, just something concrete that you can focus on instead of your problems.”

Furthermore, some of the participants commented on several aspects of the positive influence of work participation at the Clubhouse. For instance, one participant expressed a hope of reducing the amount of medication he takes thanks to his more structured daily life, and others talked about how the workday schedule made them feel more ordinary in relation to society at large, and two others reported having a more satisfying private life as a result of the structure that the Clubhouse had instilled in their lives.

Uhm, (what’s important is) a meaningful everyday life. A job to go to, and I consider this to be my job today. And then it’s about having good friends. Have some free time. Go to a party sometimes, with no kids and, right. And then … And be with my kids, do nice things … So, so (I don’t receive) any support from the Clubhouse directly to be a good mother, but (…). Well, since I have a work-ordered day, then, it probably means that I manage to be a better mother … (Maya)

Most of the participants mentioned having the option for self-improvement at the Clubhouse, such as discovering one’s work capacity, experiencing one’s own potential, taking pride in one’s work achievements, realizing new talents and interests and working on developing specific skills. In addition, one of these participants described the Clubhouse as a place for experiencing success, and as such, as an opportunity to build self-confidence and self-esteem.

Interestingly, there seemed to be marked preferences when it came to the type of work participants favoured, which appeared to extend also to the TEPs (transitional employment placements) and choice of work unit. The two poles of these preferences were clerical and kitchen work. While office work was praised as being mentally challenging and more complex and the clerical unit as being a calmer space, these were also criticized as being boring and as not showing obvious results. At the same time, kitchen work was credited as being active and producing obvious results, and hence for instant gratification, even though it was criticized as not being mentally challenging enough.

Well, all is not money, because the time we spend here on earth, it is measured, it is small. We do not know if we will ever get any more, at least as it is in my chosen, my view of life, so it is important to use it to please yourself, to please others and to enjoy your everyday life. And what better way than to cook? You know, to please others. I don’t feel like I could please others by sitting in an office, to be completely honest. (Thomas)

#### Preparing for a working life in society

Several participants said that they had become Clubhouse members to get help with getting back into the labour market. Interestingly however, none of them expressed a desire to continue in their previous professions. In fact, those who discussed this issue said that they had made their choices explicitly to avoid jobs similar to their former ones, which many of them thought had caused their recurring relapses. Moreover, one participant said he wanted to avoid the environment of his previous job, where drugs were prominent.

In addition to the participants who contemplated new career paths, several others said that participation in the Clubhouse community made them realize that they could have a career despite having a serious mental illness and use their participation in the work-ordered day as a stepping-stone towards competitive employment. They reported that the work they did for the Clubhouse community provided them with a feeling of usefulness and a sense of accomplishment, which in turn gave them hope regarding their return to the labour market. In addition, some of the participants, such as Olivia, reported receiving support to try skills they did not know they had:

(They asked me at the Clubhouse) “What do you want to work with? What are you dreaming of?” And … I’ve always dreamt of becoming an accountant. But I never dared. Because I felt I was too stupid, I wasn’t … I wasn’t good enough to be one. (…) But then a staff member said to me, “But why can’t you? You just go to evening school, you also go to college, and you go where you have to go, and then you’re done.” It was … It was that simple.

Accordingly, several participants said that being active at the Clubhouse would smooth their transition back to work from long-term sick leave or unemployment. In addition, some of these participants reported that, ideally, they would like to get a job after being a Clubhouse member for some time, even if it was “just” a part-time job, a transitional employment placement (TEP) or studies.

Notably, while several participants said that TEPs were a good opportunity, being a springboard to the labour market, a few of them noted that it was too hard to get a TEP, and most of the available TEPs were unidimensional, as explained by Thomas:

We have something called transitional employment here at the Clubhouse, and it is 100 percent office work. Or uhm, I’ll say it like that, office work one can do even without completing high school. I think we should take it a step up, because there are quite a few resources here that are smart as hell, but who, uhm, don’t like … (…) Have some that are a bit more complicated (…). And I also wish they could get more transitional employment places simply with restaurants there. Or canteens or whatever.

Beyond support in obtaining employment, several participants described many ways in which they found their Clubhouse participation useful for the labour market. Among these were the meetings at the Clubhouse, which are considered to be helpful for practising job skills such as professional communication and public speaking. In addition, Emil discussed further opportunities for learning to act and communicate professionally at the Clubhouse:

It (the Clubhouse) is for finding out how one works. What is it like to go into a canteen with lots of strangers, for example? What is it like to come to work and find a closet or room … ? Where should one put one’s bag? What, what … If one hasn’t had an office. So, all that, one would be able to try out. (…) Well, it is about getting to know themselves, to be confident in themselves.

However, not all participants expressed a desire to obtain regular, paid employment in the labour market in the future. Many reasons were given, such as feeling that one’s illness was too disabling, having been granted a disability pension and having one’s finances in order. Yet others declared themselves too old to go back to work, “having done enough for society already”, even though only a fragment of this group was over the working age (67 years in Norway).

## Discussion

A message from the participants interviewed in this study was the importance of developing a new and stronger identity, after experiencing the debilitating effects of mental illness, a notion also recognized in the literature as crucial part of being in recovery (Anthony & Mizock, 2014; Davidson et al., 2017; Deegan, 2002; Dixon et al., 2016; Norman, 2006; Solomon & Gioia, 2016). Furthermore, personal choices regarding the development of a new identity and roles are considered crucial for enhancing the recovery process, as indicated in our results as well as in previous studies on recovery (Davidson et al., 2017; Stanhope et al., 2013; Topor et al., 2020) and salutogenesis (Antonovsky, 1979, 1987a; Fekete, Kinn et al., 2020; Langeland et al., 2007).

As in previous research, our findings indicate that there is a positive link between Clubhouse members’ social networks and processes of recovery, and that the Clubhouse community is shaped by dynamic interplay between members and staff (Biegel et al., 2013; Carolan et al., 2011; Fekete, Langeland et al., 2020), where staff were described as key providers of support. As shared experience plays an important role in integrating isolated people into the community, as shown in salutogenic research (Vaandrager & Kennedy, 2017, p. 166), our results similarly showed that peer relationships were based on common experiences with ental illness.

Furthermore, research indicates that the social network of a person in recovery, including their family, care providers and peers, have a crucial impact on their recovery outcomes (Davidson & Roe, 2007; Davidson et al., 2017). For instance, a person’s social network has been identified as a key resistance resource that may help the individual cope with life challenges, thus improving their health and wellbeing (Antonovsky, 1979, 1987a; Langeland et al., 2016; Topor et al., 2011, 2020), and the quality of social support has been set out as a cornerstone of the health-promoting theory of salutogenesis (Fekete, Kinn et al., 2020; Langeland et al., 2016; Langeland & Vinje, 2016).

In terms of social support, the sense of acceptance and feeling of security at the Clubhouse community, provided a safe arena for (re)building personal identities, thus promoted recovery according to previous studies (Biegel et al., 2013; Fekete, Langeland et al., 2020; Kinn, Tanaka et al., 2018; Pernice-Duca, 2008; Tanaka & Davidson, 2015b). However, when this feeling of security was not balanced with challenges to be met, it had a negative effect on the recovery processes, an issue raised both in the present study and in previous research (Kinn, Tanaka et al., 2018; Raeburn et al., 2013). This also resonates with a previous study (Fekete, Kinn et al., 2020) that points out that overcoming challenges and solving problems give an experience of mastery and capability, could give an opportunity for enhancing positive development within the Clubhouse model as described by salutogenic theory (Langeland et al., 2016; Langeland & Vinje, 2016; Langeland et al., 2007)

Our results echo previous research that participating in meaningful activity at the Clubhouse made an important contribution to health and wellbeing (Fekete, Langeland et al., 2020; Norman, 2006; Perrins-Margalis et al., 2000; Tanaka & Davidson, 2015a; Waegemakers Schiff et al., 2008). As noted in the salutogenesis literature, work can serve both as an end and as means, to instil routines and everyday structure in a person’s life and as a source of personal growth (Langeland & Vinje, 2016). Correspondingly, the theory of salutogenesis suggests that regular meaningful occupation of any kind, such as paid or unpaid work, voluntary community activity or studies, can also be beneficial to the recovery process, as it enrichens the meaningfulness factor, the motivational element in the sense of coherence (Fekete, Kinn et al., 2020).

Obtaining regular employment is underlined as an important goal in both international and national health care and social policies (Harnois & Gabriel, 2002; International Labour Organisation, 2017; Ministry of Labour/Arbeidsdepartementet, 2013), because it promotes individuals with an opportunity to sustain themselves, gain a sense of achievement by contributing to society and experiencing self-realization. Similarly, the Clubhouse model has a prominent focus on promoting work participation for its members (Clubhouse International, 2018), thus have a preference for developing a worker role as a central goal (Kennedy‐Jones et al., 2005; Norman, 2006; Tanaka & Davidson, 2015a). In line with these aims, the majority of participants had plans regarding future employment. However, not all of the participants were willing to return to the labour market. Exploring clubhouse members’ personal reasons behind this decision, both in Norway and internationally, could reveal factors that may have an effect on these people’s vocational recovery journey.

## Conclusions

Our results indicate that vocational and social recovery in the Clubhouse context is a transformative experience: a step towards a better life and evolving into a personally preferred role in particular. Self-determination and the ability to make life choices were valued components of this process and were contingent upon the possibility of choice offered by unlimited Clubhouse membership.

While consistent with international policies the Clubhouse model focuses on possibilities enabling individuals to participate in work, our results indicate that developing a worker role was not necessarily the main goal of study participants, but rather achieve development in social aspects of their lives. Nevertheless, in line with the principles of recovery and the theory of salutogenesis, participation in meaningful work-related activities for the Clubhouse community was reported as enhancing recovery. This finding indicates that participation in meaningful activities and socializing in the community constitute all together important elements of a recovery-oriented programme.

Another recovery-promoting ingredient that emerged from this study was the provision of a wide playing field for the person in recovery, such as the unlimited membership offered by the Clubhouse model. However, self-agency and taking responsibility seemed to be key to making the best use of such an opportunity, which individuals with a poorer mental health status might not be able to achieve. Consequently, we suggest that Clubhouse communities, as well as similar PSR programmes, focus on enabling people at all stages of their recovery journeys to seamlessly join the programme, settle in and find appropriate roles. However, our study indicates that such processes should be balanced with offering greater challenge for those who need it: providing individuals opportunities to take responsibility and become empowered. These findings may be important in terms of shared decision-making in recovery-oriented services, suggesting that those persons who are able to are, given the opportunity, likely to take an interest in and actively participate in designing and conducting their own therapies/interventions.

## Limitations

While our findings are the result of a rigorous and ethical methodology, owing to the nature of qualitative research they are not intended to be representative. Rather, the aim was to enhance our knowledge on the basis of the experience and views of these participants. While we employed procedures to enhance reflexivity by carrying out the analysis and discussing the findings in a group setting, our results are not absolute (Fossey et al., 2002). Further research might uncover nuances in our findings or provide additional data. Furthermore, one must bear in mind that, due to the method of recruitment in this study, it is likely that the Clubhouse members who volunteered to participate were among those who more actively utilized the Clubhouse and who were thus probably more active in the realization of their recovery goals.
